# Management and prevention of *Neisseria meningitidis* and *Neisseria gonorrhoeae* infections in the context of evolving antimicrobial resistance trends

**DOI:** 10.1007/s10096-024-04968-8

**Published:** 2024-11-27

**Authors:** Helen S. Marshall, Jean-Michel Molina, Valérie Berlaimont, Aruni Mulgirigama, Woo-Yun Sohn, Béatrice Berçot, Shravani Bobde

**Affiliations:** 1 Vaccinology and Immunology Research Trials Unit, Women’s and Children’s Health Network and Robinson Research Institute and Adelaide Medical School, The University of Adelaide, Adelaide, Australia; 2 Université Paris Cité, INSERM UMR 944, Paris, France; 3 Department of Infectious Diseases, Saint-Louis and Lariboisière Hospitals, APHP, Paris, France; 4 GSK, Singapore, Singapore; 5 GSK, Brentford, United Kingdom; 6 GSK, Rockville, MD USA; 7 Université Paris Cité, INSERM1137, IAME, Paris, France; 8 Department of Bacteriology, French National Reference of Bacterial STI, Saint-Louis and Lariboisière Hospitals, APHP, Paris, France

**Keywords:** Meningitis, Gonorrhea, Antimicrobial resistance, Antibiotics, Vaccines

## Abstract

**Purpose:**

To describe the relationships between *Neisseria meningitidis* (NM) and *Neisseria gonorrhoeae* (NG) at genetic, population, and individual levels; to review historical trends in antimicrobial resistance (AMR); to review the treatment and preventive landscapes and explore their potential impact on AMR.

**Methods:**

A narrative literature search was conducted in PubMed, with searches restricted to 2003–2023 and additional articles included based on expertise.

**Results:**

NM and NG are closely related bacterial pathogens causing invasive meningococcal disease (IMD) and gonorrhea, respectively. NM can currently be treated with most antibiotics and generally has a wild-type susceptibility profile, whereas NG is increasingly resistant even in the first line of treatment. These pathogens share 80–90% genetic identity and can asymptomatically cohabit the pharynx. While AMR has historically been rare for NM, recent reports show this to be an emerging clinical concern. Extensively drug-resistant NG are reported globally, with data available from 73 countries, and can lead to treatment failure. Importantly, *Neisseria* commensals within the normal microbiota in the pharynx can act as a genetic reservoir of resistance to extended-spectrum cephalosporins. Novel oral antibiotics are urgently needed to treat a growing threat from antibiotic-resistant NG, recognized as a major global concern to public health by the World Health Organization. Numerous vaccines are available to prevent IMD, but none are approved for gonorrhea. Research to identify suitable candidates is ongoing.

**Conclusion:**

Holistic management of AMR in IMD and gonorrhea should couple judicious use of existing antibiotics, optimization of vaccination programs, and development of novel antibiotics and vaccines.

**Graphical abstract:**

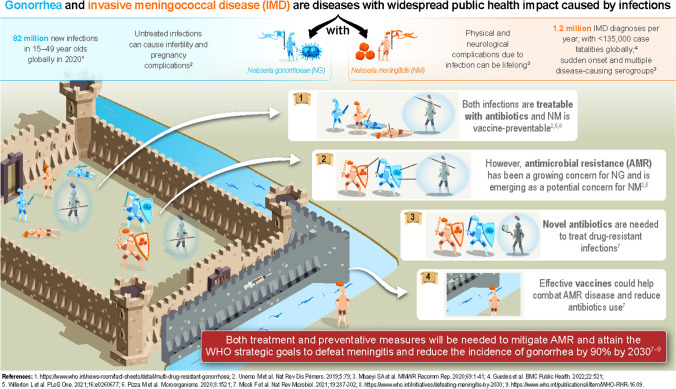

## Introduction

*Neisseria meningitidis* (NM) and *N. gonorrhoeae* (NG) are the only two bacterial species of the Neisseria genus recognized as strict human pathogens [1]. They are genetically related and mostly colonize the upper respiratory tract and genitourinary tract, respectively [2–4]. Both bacteria can inhabit the pharynx and the urogenital tract, cohabiting these anatomic niches with commensal *Neisseria* species within the normal human microbiota [5]. While closely related, the two *Neisseria* pathogens cause different clinical diseases with widespread public health impact.

NM infections can be caused by different meningococcal serogroups. Five serogroups (A, B, C, W, and Y) cause the majority of invasive meningococcal disease (IMD) cases. Although uncommon, IMD is life-threatening and a principal cause of bacterial meningitis and septicemia globally [3, 6, 7]. The case-fatality rate (CFR) of IMD ranges between 4 and 20% [8, 9], and survivors can have lifelong physical and neurological complications, as severe as amputations, hearing loss, vision loss, learning disabilities, and epilepsy [10, 11]. Clinical management is challenging due to the diverse and often nonspecific spectrum of presenting clinical features [12].

NG causes gonorrhea, which is one of the most common sexually transmitted infections (STI) in adolescents and adults worldwide [13–15]. Infections are often asymptomatic, especially in women who carry a greater disease burden than men. Indeed, if the infection is not detected or adequately treated, long-term complications in women include pelvic inflammatory disease, chronic pelvic pain, ectopic pregnancy, tubal factor infertility, and preterm pre-eclampsia. Other potential pregnancy or neonatal complications include spontaneous abortion, preterm birth, stillbirth, low birthweight, neonatal conjunctivitis, and growth retardation [13, 16, 17]. Even if treated, gonorrhea during pregnancy may lead to preterm birth [18]. In 2015 gonorrhea caused ~ 700 deaths worldwide [19]. Given the widespread public health impact of both meningitis and gonorrhea, the World Health Organization (WHO) developed global strategies aiming to defeat meningitis by 2030 [20] and reduce the incidence of gonorrhea by 90% from 2018 to 2030 [21].

Although antibiotics (e.g., β-lactams) remain the mainstay of treatment for both IMD and gonorrhea [13, 22], antimicrobial resistance (AMR) is a major and growing public health concern [23–30]. A global modelling study estimated that 4.95 million deaths (95% uncertainty interval [UI]: 3.62–6.57 million) were associated with bacterial AMR in 2019, and 25% of these deaths were directly attributed to drug resistance [31, 32]. Some reports have estimated that, by 2050, the mortality rate from AMR could be as high as 10 million deaths per year worldwide [31, 33]. Overall, global control of AMR requires a holistic approach with broad control of AMR through antibiotic stewardship, coupled with management of the specific disease [15].

The aims of this narrative review are to describe the relationship between NM and NG at genetic, population, and individual levels. It will also review the historical trends in AMR over time for both pathogens; and explore the treatment and preventative landscape, including the potential impact of vaccination and treatment on AMR.

## Relationship between NM and NG

### At the genetic level

NM and NG share up to 80–90% of their genomic identity and acquire much of their diversity by recombination [3, 34, 35]; however, they differ in their levels of diversity, how genomic variation is acquired, and their adaptive evolution of core genomes [1, 3, 36–38]. A core-pangenome analysis of NM, NG, and *Neisseria* commensal strains reported 78 gene families unique to meningococci, 452 gene families unique to gonococci, and 319 shared genetic families [1, 39]; further, four distinct protein–protein interaction clusters were identified for NM, and five for NG [39]. The specific and shared genetic elements may underlie the similarities and differences in pathogenicity and niche adaptation [1].

Importantly, NM and NG may both acquire AMR by transformation through horizontal gene transfer with *Neisseria* commensals in the pharynx (e.g., *N. cinerea*,* N. lactamica*,* N. subflava*) [40–43]. Commensal *Neisseria* species, including *N. cinerea*, might be genetic reservoirs of resistance determinants for β-lactam antimicrobials (including third-generation cephalosporin) that can be transferred to the pathogenic species of NM and NG [40]. Igawa et al. demonstrate that *N. cinerea* strains with high ceftriaxone minimum inhibitory concentrations (MICs; 1 to 2 µg/ml) possess ceftriaxone resistance-mediating *penA* sequences that can be transferred to gonococci by transformation and result in ceftriaxone and cefixime resistance [40]. This creates clinical challenges in the antibiotic management of IMD and gonorrhea, and emphasized the importance of preventative measures. Other mechanisms of horizontal gene transfer may also be important in the development of AMR and warrant further investigation [44].

While NM is not historically recognized as a significant cause of urogenital infections, the incidence of meningococcal urethritis has increased in heterosexual men in multiple locations in the US since 2015 [45]. The unique nonencapsulated clade involved in these infections may have adapted to the urogenital environment through genotypic and phenotypic changes that make it better suited to sexual transmission and colonization of the urogenital tract, including acquisition of the gonococcal denitrification pathway which facilitates anaerobic growth [45].

### At the population and individual levels

IMD and gonorrhea differ in terms of epidemiology and clinical course [1]. NM resides in the pharyngeal mucosa of healthy carriers [7, 46]. Occasionally, the bacteria invade the bloodstream and cause systemic infection [11, 12, 47]. IMD evolves rapidly and is life-threatening with a CFR ranging from 4 to 20% and lifelong sequelae in approximately 20% of survivors [8, 9, 23, 48]. The distribution of the main disease-causing serogroups varies by geographic region [49–51]. In 2019, the most frequent serogroups causing IMD were B, C, Y (US); B, C, W (South America); B, W (Europe); B (North Africa); C, W, X (Central Africa); B, W (South Africa); A, W (Central Asia); B, W (Southeast Asia); and B (Australia) [3].

The introduction of meningococcal vaccines in national immunization programs (NIPs) and for the immunization of high-risk individuals has enabled great progress in IMD prevention [52]. The global incidence of IMD is generally low, with a literature review collecting data from 77 countries between 2010 and 2019 reporting a range of 0.0–10.2 cases per 100,000 population [53]. Incidence was greatest in infants aged < 1 year (0.0–71.7 per 100,000), followed by young children aged 1–4 years (0.0–18.2 per 100,000) [53]. In some countries, secondary incidence peaks were noted in adolescents and young adults [53]. During the coronavirus disease (COVID)-19 pandemic, incidence of IMD decreased due to stringent national lockdown measures [54]. This was followed by a resurgence of disease, particularly among adolescents and young adults in Europe and Australia [55–57].

During the past 20 years, several outbreaks of IMD have occurred in Europe and North America in men who have sex with men (MSM), since meningococci can be transmitted from the urogenital tract to the pharynx, and vice versa; the pathogenic meningococci belonged mainly to an especially virulent serogroup C lineage, including isolates that could grow anaerobically in the anorectal and urogenital tracts [45].

The obligate pathogen NG causes the STI gonorrhea [13, 14]. Asymptomatic pharyngeal and rectal carriage of NG is common, and NG can cohabit asymptomatically with NM in the oropharynx, particularly in MSM [5]. Symptomatic infections can present as urethritis in men, cervicitis or urethritis in women, and in extragenital sites (pharynx, rectum, conjunctiva, and, rarely, systemically) in both sexes [13]. At the population level, high-risk groups (e.g., sex workers and their male partners or MSM) contribute to maintaining transmission of NG within communities and the development and spread of antibiotic-resistant strains [58, 59].

Gonorrhea is more widespread than IMD [60]. The WHO estimated that there were 82 million new gonorrhea infections in individuals aged 15–49 years in 2020, with a global incident rate of 19 per 1000 women and 23 per 1000 men; most cases were in the WHO African Region and the Western Pacific Region [15]. Approximately 710,000 infections were reported to the US Centers for Disease Control and Prevention (CDC) in 2021, representing a 118% increase from 2009 [61, 62]. However, the CDC estimates that there may be as many as 1.6 million new infections each year [63]. As no specific vaccine against gonorrhea is currently available, prevention relies on promoting safe sexual behaviors and STI testing enabling prompt diagnosis and treatment initiation [13].

## Global trends in AMR

### Emergence of AMR in NM and NG

In the 1960s, NM and NG were both highly susceptible to penicillin [64]. Following decades of antibiotic exposure, the MICs of penicillin against NG increased markedly [64]. A similar evolutionary trend was observed for *Neisseria* commensals forming part of the normal human microbiota although the MIC of commensal *Neisseria* was already higher than that of NG and NM in the past [64]. Genetic transformation from resistant strains of the commensal *N. cinerea* in the pharynx has been suggested as a probable source of β-lactam resistance in both NM and NG [64]. This suggests that commensal *Neisseria* species can exchange genetic information, leading to AMR across species. Consequently, MIC surveillance of *Neisseria* commensals could be monitored as a warning sign of a potentially increased risk of the emergence of AMR in *Neisseria* pathogens [64].

Evidence of AMR in NG has been accumulating globally over the past eight decades [13, 65], leading to the recognition of drug-resistant NG as a major public health threat by the CDC [30] and the WHO [66]. Of note, the CDC reports that approximately half of all NG infections are resistant to at least one antibiotic and that only one recommended treatment remains for gonorrhea [67]. NG has acquired or evolved AMR via multiple mechanisms of resistance, the effects of which are cumulative [68]. Additionally, in the absence of antimicrobials these AMR mechanisms do not appear to lower the biological fitness of NG, as is typically seen in other resistant bacterial strains, and some AMR determinants, such as gyrA mutations, might even enhance the fitness of certain NG strains [68]. While AMR in NM has historically been uncommon, evidence of new cases has recently emerged in North America, Europe, and Asia [9, 22, 24–26, 69, 70].

In the USA, 500 mg ceftriaxone is currently the last treatment option for gonorrhea after removal of ciprofloxacin (in 2006) [71], cefixime (in 2012) [72], and azithromycin (in 2020) [73, 74] from the US CDC guidelines due to concerns over resistance. Current US guidelines advocate single-dose ceftriaxone with the addition of a course of doxycycline if chlamydial infection has not been excluded [75]. Current European guidelines (2020) also recommend high-dose ceftriaxone plus azithromycin in adults with uncomplicated gonorrhea, or in well-controlled settings, ceftriaxone monotherapy [76, 77]. Monitoring resistance will be particularly important until new drugs or vaccines become available [78]. Overall, the number of effective oral and parenteral antibacterial treatments has decreased, despite prevention programs from public health authorities and recommendations to use condoms [13, 65, 79]. This led the US CDC and the WHO to recognize drug-resistant NG as a major worldwide threat to public health [30, 66].

β-lactams, a class that includes penicillins, cephalosporins, carbapenems, and monobactams, are the main antibiotics for the treatment of both IMD and gonorrhea [13, 22]. Currently, at most centers, empirical therapy for suspected acute bacterial meningitis comprises parenteral administration of the third-generation cephalosporin ceftriaxone (or cefotaxime) [80–82]. Typically, parenteral ceftriaxone or cefotaxime is continued after the identification of NM, and a single oral dose of ciprofloxacin 500 mg may be added for patients not treated with ceftriaxone [81, 82]. Ciprofloxacin, rifampin, and ceftriaxone can be used for the chemoprophylaxis of close contacts [22, 83]. Rare reports of AMR involving these currently recommended treatments have been published since the 1990s [25, 84].

There is evidence that β-lactam resistance is increasing worldwide in both NM and NG [85, 86]. In 2012, a genetic variant in a penicillin-binding protein (i.e., *penA327* allele) originated in NG was observed in NM strains with decreased susceptibility to third-generation cephalosporins [85, 87, 88]. A structurally similar variant (*penA1C* allele) currently found only in NG confers extensive resistance to third-generation cephalosporins, raising the possibility of NM strains with resistance to third-generation cephalosporins in the future [85]. Additionally, there has also been recent international spread of ceftriaxone-resistant NG strains linked to the *penA60* allele [89–91].

### AMR trends for NM

Resistance of NM to sulfonamides, penicillin, and chloramphenicol was reported in the 1970s and 1980s [92–94], but historically, AMR reports have been rare [23, 69]. Penicillin resistance in NM generally remains at < 5% worldwide, although a few studies reported rates of non-sensitivity to penicillin of up to 27% [95], suggesting a potential reduction in the suitability of penicillin as a treatment for IMD [96]. In Egypt, a study reported more than 40% of NM isolates as resistant to penicillin [97], and in Ethiopia, more than 30% of NM isolates were reportedly resistant to ampicillin [98]. Moreover, in India and Southeast Asia, high rates (in some cases > 70%) of quinolone resistance in NM have been reported [85, 99].

Importantly, and, somewhat surprisingly, given recent periods of social isolation due to COVID-19, there have been recent reports of drug-resistant NM strains in the UK [22], the US [23, 69], China [24], Japan [25], and Southeast Asia [26]. In the US, AMR was reported among meningococcal serogroup Y (MenY) isolates containing a β-lactamase gene (*bla*_ROB−1_) associated with penicillin resistance (22 cases) or penicillin and ciprofloxacin resistance (11 cases) [23]; of relevance, MenY isolates caused recent outbreaks with CFR up to 27% [23, 69]. In France, testing of various meningococcal isolates (primarily serogroups B [50%], C [18%], W [17%], and Y [14%]) revealed a general increase in intermediate susceptibility to penicillin, from 36% of isolates in 2017 to 58% in 2021 [100].

A systematic review and meta-analysis of 24 studies worldwide published between 2000 and 2020 showed low resistance rates among NM (1–3.4%) to ceftriaxone, cefotaxime, ciprofloxacin, and rifampin and a higher rate to penicillin (27.2%) [95]. Additionally, AMR increased in the second half versus first half of this two-decade period [95]. Based on these data, NM was recently flagged as a reemerging pathogen with increasing AMR and an emerging clinical concern [101].

### AMR trends for NG

For NG, AMR has been an increasing concern over the past eight decades, with resistance to sulfonamides and penicillin observed since the 1940s [13, 65]. This expanded to spectinomycin in the 1960s, tetracycline in the 1980s, and ciprofloxacin, cefixime, and azithromycin in the 1990s [13, 65].

In the WHO Asia-Pacific region in 2011–2016, the percentage of locations reporting > 5% of gonococcal isolates with decreased susceptibility to ceftriaxone increased from 14.3 to 35.3%, and the percentage of locations reporting > 5% of gonococcal isolates with resistance to azithromycin increased from 14.3 to 38.9% [102]. A review of AMR surveillance data from Africa found that the overall extent of surveillance is low, with data available from just 13 of 54 African countries, and that suboptimal protocols are frequently used to conduct antimicrobial susceptibility testing [103]. NG with very high rates of resistance to ciprofloxacin, tetracycline, and penicillin are present in all regions of sub-Saharan Africa; resistance to azithromycin has been observed in all regions, whereas resistance to cefixime or ceftriaxone has been less commonly observed in West Africa [104]. A systematic review of AMR patterns in Latin America and the Caribbean found widespread resistance to penicillin, tetracycline, and ciprofloxacin; variable levels of resistance to azithromycin (up to 32%, Brazil 2014–2017); and infrequent resistance to extended-spectrum cephalosporins [105]. It is important to note that study designs, methods for performing antimicrobial susceptibility testing, and MIC breakpoints used to interpret the results (e.g., Clinical and Laboratory Standards Institute or EUCAST breakpoints) may vary across studies [104, 106].

Extensively drug-resistant strains of NG have now been reported globally [1, 27–29, 107]. A WHO surveillance program involving data submitted from 73 countries during 2017–2018 showed decreased susceptibility or resistance of NG strains to ceftriaxone (in 21/68 countries; 31%), cefixime (24/51 countries; 47%), azithromycin (51/61 countries; 84%), and ciprofloxacin (70/100 countries; 100%) [108].

The first treatment failure with ceftriaxone was reported in Japan in 2009 [13, 65], and in the past 10 years, treatment failures for ceftriaxone (± azithromycin or doxycycline) have been reported in Australia, France, Japan, Slovenia, Sweden, and the UK [15]. These treatment failures with currently recommended antibiotics indicate the potential for a period of untreatable gonorrhea in the near future [86, 109]. In the US, the Massachusetts Department of Public Health recently reported a novel gonorrhea strain with resistance (one case) or a decreased response (another case) to five antibacterial classes; however, high-dose ceftriaxone treatment was successful in both cases [110].

### New antibiotics in development for gonorrhea

Given the growing concerns regarding increased AMR and the reports of treatment failures with the currently recommended antibiotics [23–30], novel antibiotics are urgently needed for the treatment of drug-resistant gonorrhea [111].

Two novel first-in-class antibiotics are currently being investigated in phase 3 studies: gepotidacin and zoliflodacin are the first triazaacenaphthylene and spiropyrimidinetrione, respectively [112]. Gepotidacin selectively inhibits bacterial DNA gyrase (GyrA) and topoisomerase IV (ParC) by a distinct binding site, unique from other therapeutics [113, 114]. It is likely that mutations in both enzymes would be needed for resistance to occur and surveillance of susceptibility to gepotidacin (both phenotypically and genomically, and especially regarding GyrA A92 and ParC D86) will become imperative [115–117]. Zoliflodacin inhibits bacterial type II topoisomerases by targeting the GyrB protein which is different to the mechanism of action of fluoroquinolones, which target the GyrA and ParC proteins [118]. Evidence from phase 2 and 3 trials conducted in the US suggests that gepotidacin and zoliflodacin are highly effective for urogenital infections caused by NG (Table 1) [119–121]. In the phase 2 randomized study, a single oral 1500 or 3000 mg dose of gepotidacin produced an overall microbiologic cure rate of ≥ 95% [121]. While three microbiologic failures and two cases of resistance development to gepotidacin were reported in the phase 2 study, additional pharmacokinetic/pharmacodynamic modeling predicted that use of a higher dose for the phase 3 trial (two 3000 mg doses given 10–12 h apart) would provide better efficacy and limit the potential for resistance development [121–123]. It should be noted that treatment failures for gepotidacin and zoliflodacin have been reported at the oropharyngeal site, an important site of infection in MSM and sex workers, although data is limited as only two cases of pharyngeal NG were reported in the phase 2 study [121, 124]. At the time of publication, no cases of treatment failure at extragenital sites have been reported in phase 3 trials. No treatment-limiting adverse events (AEs) were observed for either dose, and most reported AEs were gastrointestinal, as is frequently the case with antibacterial therapies [121, 125].

**Table 1:** Efficacy of gepotidacin, zoliflodacin, and ceftriaxone against uncomplicated urogenital gonorrhea: results of randomized phase 2 and phase 3 trials

Phase and reference*	Study design	Drug (*N* randomized)	Confirmed NG infection, *n*	Microbiologic eradication, % (*n*)	Pre-defined non-inferiority criterion	Treatment difference (%, 95% CI)
Phase 3 [119, 126]	R, MC, NI	Gepotidacin 3000 mg PO × 2 (202)	187	92.6 (187)	Lower limit of the two-sided 95% CI >–10.0%	–0.1 (–5.6, 5.5)
		Ceftriaxone 500 mg IM plus azithromycin 1000 mg PO × 1 (204)	186	91.2 (186)		
Phase 3 [130]	R, MC, NI	Zoliflodacin 3000 mg PO × 1 (621)	621^a^	90.9^b^	12%	5.31 (1.38, 8.65)
		Ceftriaxone 500 mg IM plus azithromycin 1000 mg PO × 1 (309)	309^a^	96.2^b^		
Phase 2 [121]	R, MC	Gepotidacin 1500 mg PO × 1 (53)	30^c^	29 (97)		NA
		Gepotidacin 3000 mg PO × 1 (53)	39^c^	37 (95)		
Phase 2 [120]	R, MC	Zoliflodacin 2000 mg PO × 1 (72)	57^d^	55 (96)	−−−−−−−−−−−−−−	NA
		Zoliflodacin 3000 mg PO × 1 (67)	56^d^	54 (96)	−−−−−−−−−−−−−−	
		Ceftriaxone 500 mg IM × 1 (41)	28^d^	28 (100)		

*CI* confidence interval; *IM* intramuscular, *micro-ITT* microbiologic intent-to-treat; *MC* multicenter; *NG Neisseria gonorrhoeae*; *NI* non-inferiority; *PO* oral; *R* randomized

*Studies listed are distinct, unrelated trials, and thus comparisons should not be made between them

^a^Key inclusion criteria for this study were: age ≥ 12 years; signs and symptoms of uncomplicated urethral or endocervical gonorrhea; and/or culture, Gram stain or nuclear acid amplification test positive for NG within 14 days prior to screening; and/or unprotected sexual contact with confirmed infected partner within 14 days prior to screening

^b^N numbers not reported in reference

^c^The microbiologically evaluable population included all randomized participants who had NG isolated from baseline cultures of urogenital swab specimens, received either dose of gepotidacin, and returned for test of cure

^d^The micro-ITT population included all randomized participants with NG at urethral or cervical sites at enrollment

The global randomized phase 3 EAGLE-1 trial (NCT04010539; *N* = 620) showed that gepotidacin (oral, two doses of 3,000 mg) was non-inferior to ceftriaxone (intramuscular, 500 mg) plus azithromycin (oral, 1000 mg) in adolescent and adult participants with uncomplicated urogenital NG, with microbiological success rates (determined 3–7 days post treatment) of 92.6% and 91.2%, respectively [119, 126]. In the microbiologically-evaluable population, the success rate of gepotidacin was 100% for both treatment groups [119]. No isolates from any site developed resistance to gepotidacin, and safety results were consistent with phase 1/2 data [119]. The positive results from the EAGLE-1 trial highlight the future potential of gepotidacin as a new oral treatment for uncomplicated urogenital NG. Recently, gepotidacin (oral, two daily 1500 mg doses for five days) was reported to be non-inferior (EAGLE-2, NCT04020341; *N* = 1531) or superior (EAGLE-3, NCT04187144; *N* = 1605) to nitrofurantoin (oral, two daily 100 mg doses) in adolescent and adult female individuals with uncomplicated urinary tract infections [127].

Gepotidacin had consistent in vitro activity against NG when tested against 252 clinical isolates and international reference strains, with no evidence of target-specific cross resistance with other antibiotics [128]. The presence of the ParC D86N mutation associated to the GyrA S91F + GyrA D95G mutation, which confers high level of resistance to fluoroquinolones, was associated with increased MICs to gepotidacin [128].

Zoliflodacin was also effective for urogenital infections in a randomized phase 2 study. Zoliflodacin (oral, 2000 or 3000 mg doses) produced microbiologic cure rates of 96% with both doses compared with 100% achieved with ceftriaxone (intramuscular, 500 mg) [120]. Most reported AEs were gastrointestinal [120]. In a recently completed phase 3 noninferiority study, zoliflodacin (oral, 3000 mg) was compared with ceftriaxone (intramuscular, 500 mg) plus azithromycin (oral, 1000 mg) in adolescents and adults with uncomplicated gonorrhea (ClinicalTrials.gov identifier: NCT03959527; *N* = 930) [129]. According to a press release and data presented at ECCMID 2024, the efficacy of zoliflodacin was noninferior to the comparator using a pre-specified 12% margin (treatment difference: 5.31% lower for zoliflodacin vs. comparator, 95% CI 1.38, 8.65%), and the safety profile was also similar to the comparator [129]. The success rate for zoliflodacin was 96.8% in the microbiologically-evaluable population [130].

When evaluated against clinical NG isolates from Europe [131], and from Thailand and South America [132], in vitro antibacterial activity of zoliflodacin was consistent, and no evidence of cross-resistance between zoliflodacin and any other antimicrobial was observed. The zoliflodacin target GyrB was highly conserved and no zoliflodacin *gyrB* gene resistance mutations were detected in the European study [131].

## Impact of vaccination in reducing or preventing AMR

Lessons learned from studies evaluating different vaccines support the notion that vaccines could reduce disease burden and antibiotics use [133–135]. Firstly, vaccines reduce both prevalence of the resistant pathogen and antibiotic use [133]. An example is provided by vaccination against *Streptococcus pneumoniae*, with a few studies indicating that reduced carriage of, and infections due to, *S. pneumoniae* in vaccinated individuals led to markedly decreased antibiotic prescribing and reduced circulation of resistant *S. pneumoniae* strains; therefore, herd immunity may be a principal factor in decreasing the circulation of pneumococci with AMR [133–135]. In the US, rates of antibiotic-resistant invasive pneumococcal infections decreased in children and adults after introduction of a 7-valent conjugate vaccine in 2000–2004 [136]. In Finland, use of a 10-valent pneumococcal conjugate vaccine (PCV10) significantly reduced the proportion of multidrug-resistant *S. pneumoniae* isolates from 22 to 6%, from 2009 to 2014, in children aged < 5 years [135].

A systematic literature review of studies published in 2008–2017, primarily in France and the US, evaluated the impact of routine infant immunization with PCV10 or PCV13 on AMR in children and adults. This analysis concluded that vaccination of children with PCV13 was associated with a reduced incidence of AMR otitis media and nasopharyngeal carriage in children and of AMR invasive pneumococcal disease in both children and adults [137].

Consistent with these findings, a systematic review of data from 559 studies of pediatric isolates from 104 countries showed decreases in pneumococci resistant to penicillin, trimethoprim/sulfamethoxazole, third-generation cephalosporins, macrolides, and tetracycline over 10 years after implementation of any PCV immunization program [138].

Vaccination more broadly may also have an indirect effect on AMR by preventing viral infections and secondary bacterial superinfections that may occur in individuals infected with a virus, as observed for influenza vaccination [133, 139].

### Vaccination to prevent IMD

Vaccines protecting against the main disease-causing meningococcal serogroups have been introduced worldwide (Table 2), resulting in a reduced overall disease burden [3, 48, 140]. Since the UK first introduced MenC vaccination in the infant NIP in 1999, many countries have followed, using different schedules and targeting different age groups [52]. In the UK, Australia, and other countries, quadrivalent conjugate vaccines against meningococcal serogroups A, C, W, and Y (MenACWY) have now replaced or will replace monovalent vaccines against serogroups C and A for all adolescents and young adults [52, 80, 141, 142]. Post-licensure data from many countries has shown demonstrable positive impacts through both direct and herd protection [143]. MenACWY vaccines can reduce incidence of IMD even in high-exposure groups, with a recent study of soldiers in South Korea demonstrating a reduction of 88% in IMD incidence following vaccination [144]. Two protein-based vaccines against serogroup B are available, 4CMenB and MenB-FHbp. The latter is not currently included in any NIPs but is approved for individuals aged 10–25 years in the US, Canada, and Brazil and those aged ≥ 10 years in Australia, Europe, and a number of other countries [52]. 4CMenB is the only MenB vaccine licensed for use in infants, with vaccination in infants recommended and publicly funded in the UK and several European countries. Additionally, vaccination in adolescents is recommended in the Czech Republic, South Australia, and recently Queensland, Australia. In the three years following the introduction of the 4CMenB vaccine program in England, a continued positive effect against group B IMD was observed [145]. Real-world data has also shown that the protective effects of 4CMenB may extend to other subgroups, with effectiveness seen against MenW disease in the UK [146, 147].

**Table 2:** Currently available vaccines against *Neisseria meningitidis* [52, 80, 183–185]

Vaccine formulation and name	Serotypes covered	Trade name	Indication^a^
*Conjugate vaccines*			
MenA	A	*MenAfriVac* (Serum Institute of India)	Active immunization of children aged 3–24 months against NM group A
MenC	C (Hib)	*Menitorix* (GSK); *Menjugate* (GSK); *NeisVac-C* (Pfizer); *Meningitec* (Nuron Biotech)	Active immunization of children aged 2–24 months against Hib and NM group C^b^
MenACWY	ACWY	*Menactra* (Sanofi); *MenQuadfi* (Sanofi); *Menveo* (GSK); *Nimenrix* (Pfizer)	Active immunization of individuals aged 9 months to 55 years against NM serogroups A, C, Y, and W^c^
*Polysaccharide vaccine*			
*Protein-based vaccines*			
4CMenB	B	*Bexsero* (GSK)	Active immunization of individuals aged ≥ 2 months against NM group B^d^
MenB-FHbp	B	*Trumenba* (Pfizer)	Active immunization of individuals aged 10–25 years against NM serogroup B
OMV meningococcal BC	BC	*VA-Mengoc-BC* (Finlay Institute)	Active immunization from 3 months of age against NM serogroup B and C^e^
*Combination conjugate and protein-based vaccine*			
MenACWY-TT/MenB-FHbp	ABCWY	*Penbraya* (Pfizer)	Active immunization from 10 to 25 years of age against NM serogroups A, B, C, W, and Y

*EMA* European Medicines Agency, *FHbp* meningococcal surface protein factor H-binding protein, *Hib Haemophilus influenzae* type b, *Men* meningococcus, *NM* Neisseria meningitidis, *OMV* outer-membrane vesicle

^a^Information from US package insert, unless otherwise stated

^b^From UK Summary of Product Characteristics for combined Hib and meningococcal group C vaccine (*Menitorix*)

^c^From US Prescribing Information for *Menactra*

^d^From EMA Summary of Product Characteristics for *Bexsero*

^e^From *VA-Mengoc-BC* package insert (Finlay Institute, Cuba)

Prevention of IMD with meningococcal vaccines could contribute to a reduction in antibiotic use and AMR. Interestingly, it has been demonstrated that influenza infections contribute to a population risk of IMD and that vaccination against seasonal influenza may reduce the risk of IMD and related antibiotic use [148]. Moreover, the 4th Summit Meeting of the Global Meningococcal Initiative highlighted the decrease in IMD infections as well as vaccinations during COVID-19 (with an increase in infections observed post-COVID [54]) including that pre-emptive use of broad-spectrum antibiotics might have promoted AMR and catchup IMD vaccination could help compensate the reduction in vaccination [54].

Based on the recent reports of AMR in NM and the recognition that this is an emerging clinical concern, using a combination of these approaches, especially improving prevention via vaccination, will be important in future decades [101]. Accordingly, because of the high CFR and severe sequelae that can result from NM infection, despite prompt antibiotic treatment [140], vaccination is the most important aspect of the WHO-led strategy aiming to defeat meningitis by 2030 [20, 80].

### Vaccination to prevent gonorrhea

There are currently no approved vaccines for prevention of gonorrhea [149], an important global barrier to the management of this disease. Traditionally, several factors hindered the development of specific gonococcal vaccines: the biological diversity and high plasticity of antigens in NG strains, making the selection of relevant vaccine antigens difficult; the lack of an immunoprotective response after NG infection, limiting the availability of biologic indices of immunoprotection; and the lack of animal models of NG infection that accurately mimic the clinical setting of human infection [150–152]. Failure of at least one vaccine candidate trialed in humans against the pilin protein was attributable to the significant antigenic variability demonstrated by NG [153]. Existing animal models also present challenges due to NG being a strictly human pathogen. While female mouse genital tract infection models have allowed for candidate antigenic screening there are still limitations in their ability to accurately reflect infection dynamics in humans [153].

Bacterial outer-membrane vesicles (OMVs) as potential vaccine antigens have stimulated much recent research interest [150], as have novel liposomal and nanoparticle formulations [152]. Observational studies have reported 31–47% effectiveness of serogroup B OMV-based meningococcal vaccines against gonorrhea (Table 3) [154–160]. A cohort and case-control study evaluated the impact of the South Australia 4CMenB vaccination program on serogroup B meningococcal disease and gonorrhea [154]. Two-dose vaccine effectiveness against serogroup B IMD was 94.2% (95% CI 36.6–99.5; screening method) in children and 100% in adolescents and young adults [154]. Estimated two-dose vaccine effectiveness against gonorrhea in adolescents and young adults was 32.7% (95% CI 8.3–50.6) [154].

**Table 3:** Effectiveness of serogroup B OMV meningococcal vaccines against gonorrhea based on observational studies

Country and study	Vaccine	Population and age range	Study design	Control	Observation period post vaccination, years	Effectiveness (95% CI)
New Zealand [186]	MeNZB^a^	Adolescents and young adults, 15–30 years old	Case-control	Individuals with chlamydia	1, 3, and 6 months	31% (21, 39)
US [155]	4CMenB	Adolescents and young adults, 16–23 years old	Case-control	Individuals with chlamydia	2	40%^c^ (23, 53)
Australia [154, 158]	4CMenB	Adolescents and young adults, 15–20 years old	Cohort and case-control	Individuals with chlamydia	3	33.2% (15.9, 47.0)
US [156]	4CMenB	Adolescents and young adults, 15–30 years old	Matched cohort	MenACWY	1.9 (1.16–2.97)^b^	46%^d^
Italy [157]	4CMenB	MSM living with HIV, ≥ 18 years old	Case-control	Individuals with ≥ 1 STI other than gonorrhea	3.8 (2.1–4.3)^b^	42% (6, 64)
US [160]	4CMenB	18–29 years old	Case-control	Non–OMV-based MBV recipients	1 month to 2 years	47% (13, 68)

*CI* confidence interval, *HIV* human immunodeficiency virus, *MBV* meningococcal group B vaccine, *Men* meningococcus, *MSM* men who have sex with men, *OMV* outer-membrane vesicle, *STI* sexually transmitted infection

^a^The MeNZB vaccine was developed to control a serogroup B epidemic and is no longer available; the new generation 4CMenB vaccine uses the same OMV as MeNZB

^b^Reported as median (range)

^c^Reported as adjusted prevalence ratio

^d^Reported as hazard ratio

Although the mechanisms by which meningococcal vaccines may protect against gonorrhea are not yet well understood, these mechanisms may involve the homology of OMVs and recombinant *Neisseria* heparin-binding antigen (NHBA) proteins present in both NM and NG and included in meningococcal vaccines; further, OMV-induced antibodies recognize gonococcal proteins and NHBAs [3, 151, 155, 159, 161]. In addition, antibody activity studies showed that 4CMenB elicits antigonococcal NHBA antibodies [151, 162]. A reduction in pharyngeal carriage of unencapsulated meningococci suggests absence of a capsule as seen with gonococci may facilitate outer membrane protein exposure and susceptibility to bacterial killing [163]. Overall, clearer definition of mechanisms responsible for the antigonorrheal efficacy of meningococcal vaccines, including characterization of the genomics and proteomics of *Neisseria* species [164], will facilitate the ongoing development of clinically effective vaccines for gonorrhea [37, 159, 165].

Preclinical, clinical, and proteomic studies evaluating suitable vaccine candidates are ongoing, including a randomized clinical trial evaluating efficacy and immunogenicity of an investigational vaccine based on the generalized modules for membrane antigens (GMMA) technology [13, 159, 166].

The efficacy and safety of 4CMenB for the prevention of gonorrhea have been evaluated in several completed and ongoing studies (Table 4), including phase 2 and phase 3 clinical trials [167–175].

**Table 4:** Prospective and retrospective studies of serogroup B OMV-based meningococcal vaccines for the prevention of gonorrhea

Trial name, registration, design	Country	Study population and age, years	No. of participants	Vaccine	Comparator	Follow-up	Status
*Randomized phase 3 studies*							
ANRS174 DOXYVAC; NCT04597424; mc [171, 175]	France	MSM on PrEP or HIV+, ≥ 18	556	4CMenB	No vaccine^a^	14 months	aHR 0.78 (95% CI 0.60–1.01); *p* = 0.061 [175]) Trial completed
GoGoVax; NCT04415424; db, mc, pc [167]	Australia	GBM + on PrEP or HIV+, 18–50	730	4CMenB	Placebo	2 years	Ongoing
MenGO; ACTRN12619001478101; [168]	Australia	MSM on PrEP, ≥ 18	130	4CMenB	No vaccine	2 years	Ongoing
NCT05766904; db, pc [170]	China	MSM, ≥ 18	150	4CMenB	Placebo	2 years	Ongoing
*Randomized phase 2 studies*							
NCT04350138; mc, ob, pc [169]	US, Thailand	Men and women, 18–50	2200	4CMenB	Placebo		Ongoing^b^
NCT05294588; human challenge db, cr [172]	US	Men, 18–36	120–140	4CMenB	Flu/TDP	NA	Ongoing
*Observational studies*							
NCT05873751; co [174]	US	Men and women, 15–30		MenB-FHbp + MenACWY	MenACWY only	PharMetrics Plus database from 01 Jan 2016 to 31 Dec 2022	Ongoing
NCT04398849; “*B part of it NT*”. Immunisation for adolescents against serious communicable diseases. *Cohort and case-control study* [173]	Northern Territory, Australia	Adolescents, 14–19	2649 recruited, recruitment completed	4CMenB	No comparator	Follow-up 3 years	Undergoing analysis

*aHR* adjusted hazard ratio, *CI* confidence interval, *co* cohort, *cr* crossover, *db* double-blind, *FHbp* meningococcal surface protein factor H-binding protein, *Flu/TDP* combined quadrivalent influenza and tetanus/diphtheria vaccine, *GBM +* men (cis and trans), trans women, and non-binary people who have sex with men, *HIV* human immunodeficiency virus, *mc* multicenter, *Men* meningococcus, *MSM* men who have sex with men, *NA* not applicable, *ob* observer-blind, *OMV* outer-membrane vesicle, *pc* placebo-controlled, *PrEP* pre-exposure prophylaxis with tenofovir disoproxil fumarate/emtricitabine

^a^Participants are randomized in a 2 × 2 factorial manner to one of four study groups in which they receive either 4CMenB or no 4CMenB and either doxycycline or no doxycycline single dose for post-exposure prophylaxis

^b^Due to be completed in the final quarter of 2025

A recently completed phase 3 randomized controlled trial (RCT, DOXYVAC) evaluated the impact of 4CMenB on time to a first episode of NG infection in MSM receiving post-exposure prophylaxis (PrEP) with a history of STI [175]. In the final analysis, although fewer vaccine than placebo recipients developed NG during a median follow-up of 14 months, the difference was not statistically significant (adjusted hazard ratio: 0.78 [95% CI: 0.60–1.01]; *p* = 0.061). Analysis of secondary efficacy outcomes (cumulative incidence of gonorrhea and of symptomatic gonorrhea) provided similar results [175]. Thus, the data available to date on the effect of 4CMenB on gonorrhea is heterogeneous and additional data from ongoing studies (Table 4) may shed further light on the effect of 4CMenB against NG.

The safety and reactogenicity of an investigational vaccine (GSK4348413A) based on NG GMMAs are being evaluated in a phase 1/2, first-in-human, proof-of-concept study [166]. GMMAs are OMVs with an over-vesiculating phenotype and reduced lipopolysaccharide endotoxicity [176]. Preclinical and proteomic studies aimed at identifying suitable vaccine candidates are also ongoing [13, 159].

Importantly, modeling data suggest that a vaccine with modest efficacy against gonorrhea could have a marked influence on disease prevalence [159]. One such study in England, which assumed 31% vaccine efficacy against gonorrhea and 85% vaccine uptake, calculated that adolescent 4CMenB vaccination, together with MenACWY vaccination, could potentially avert 25% (95% credible interval: 17–33) of heterosexual incident gonorrhea infections over 70 years; catch-up and booster vaccinations could also enhance impact of the vaccination program and reduce the risk of additional AMR [177]. Another model predicted that vaccination of 100,000 heterosexual individuals at age 13 years (for a non-waning vaccine with 50% efficacy) would reduce the prevalence of gonococcal infection by ≥ 90% after 20 years; the corresponding predicted decrease in the prevalence of gonococcal infection for a non-waning vaccine with only 20% efficacy was 40% [178]. Finally, a model simulating gonorrhea transmission from 2008 to 2030 (using surveillance data from 2008 to 2017 for calibration) predicted that, if all MSM who attend sexual health clinics were vaccinated against gonorrhea with a vaccine with 31% efficacy over 2–4 years, the occurrence of gonorrhea in the vaccinated population would decrease by 45% in 2030 (even if untreatable gonococcal infections emerge) [179].

Overall, these estimates of the predicted impact that a gonorrhea vaccine could have on disease incidence taken together with the lessons learned from other vaccines on AMR reduction (see *Impact of vaccination in reducing or preventing AMR*) suggest that vaccination programs against gonorrhea would likely bring a substantial contribution to mitigating AMR in NG.

## Conclusions

IMD and gonorrhea have a widespread public health impact, which the WHO set out to tackle with global strategies aiming to defeat meningitis by 2030 [20] and to reduce the incidence of gonorrhea by 90% from 2018 to 2030 [21]. In this review, we described the relationship between NM and NG, the growing trends in AMR for those bacteria, and the impact of both antibiotics and vaccines on AMR.

NM and NG are closely related bacterial species that can cause disease but also asymptomatically cohabit anatomic niches with *Neisseria* commensals within the normal microbiota. Antibacterial-resistant *Neisseria* commensals (e.g., *N. cinerea*) can transfer genetic material to NM and NG in the pharynx and promote AMR [40–42].

While historically rare, recent reports of AMR in NM have pointed to an emerging clinical concern, especially as a few cases of potential treatment failure with ceftriaxone have been flagged [23, 25, 69, 84, 90]. AMR in NG has been a growing public health issue in the past eight decades, with multiple reports of treatment failures with the currently recommended treatments [13, 65]. The number of drugs available to treat gonorrhea is decreasing and novel antibiotics to treat emerging drug-resistant strains of gonorrhea are urgently needed. New candidates to treat gonorrhea which are under development include anti-gonococcal peptides [180] and fatty acids [181] which hold the potential to treat without development of AMR. Future research directions should also focus on understanding the genetic interactions between NM and NG, and how this knowledge can be leveraged to develop more effective AMR interventions.

In the current climate of increasing AMR, coupled with evidence that vaccination could reduce AMR disease, it is clear that tandem disease-management strategies incorporating both novel antibiotics and vaccines will be needed to attain the WHO strategic goals [20, 21, 133].

Development of an effective vaccine protecting against gonorrhea is estimated to provide substantial public health benefits, with candidates currently being evaluated in preclinical, clinical, and proteomic studies [13, 37, 159, 165–172, 175]. These include the GMMAs-based gonorrhea vaccine currently under investigation. Results of randomized clinical trials evaluating the efficacy of serogroup B OMV meningococcal vaccines against gonorrhea are awaited [182]. In addition, novel antibiotics (i.e., gepotidacin, zoliflodacin) are currently in late-stage development and, if approved, will be the first new classes of small molecule antibiotics to become available for the treatment of gonorrhea in over a decade. Overall, the holistic management of AMR in IMD and gonorrhea will have to couple judicious use of existing antibiotics with optimization of vaccination programs, as well as development of novel vaccines and antibiotics.

## Methods

### Literature search

A literature search was conducted in PubMed for full-text publications in English language using the following separate search strings: (‘*Neisseria meningitidis’* OR ‘invasive meningococcal disease’) AND (‘*Neisseria gonorrhoeae*’ OR ‘*Neisseria gonorrhea*’ OR ‘gonorrhea’); (‘*Neisseria meningitidis*’ OR ‘invasive meningococcal disease’) AND (‘*Neisseria gonorrhoeae*’ OR ‘*Neisseria gonorrhea*’ OR ‘gonorrhea’) AND ‘vaccines’; (‘*Neisseria gonorrhoea*’ OR ‘*Neisseria meningitidis*’) AND ‘antimicrobial resistance’; (‘*Neisseria meningitidis*’ OR ‘invasive meningococcal disease’ OR ‘*Neisseria gonorrhoeae*’ OR ‘Neisseria gonorrhea’ OR ‘gonorrhea’) AND ‘antimicrobial resistance’ AND ‘impact of vaccines’. Relevant congress abstracts and presentations and studies from Clinicaltrials.gov were also included in this review.

The searches were restricted to 2003–2023 and limited to clinical trials, meta-analyses, RCTs, reviews, and systematic reviews. Articles were selected for inclusion based on titles and abstracts. Additional articles were included by the authors based on expertise.

### Data Availability

Data sharing is not applicable to this article as no new datasets were generated or analyzed for this publication.
